# The Role of Plasmapheresis in Severe Leptospirosis Refractory to Standard Therapy: A Case Report and a Comprehensive Literature Review

**DOI:** 10.7759/cureus.63682

**Published:** 2024-07-02

**Authors:** Rizwan Ullah, Karim Al Harakeh, Fazeel Hussain, Syed Bazilah Mehmood Rufai, Waqar Khan

**Affiliations:** 1 Internal Medicine, Hayatabad Medical Complex Peshawar, Peshawar, PAK; 2 Internal Medicine, American University of Antigua, St. John's, USA; 3 Internal Medicine, Bangladesh Medical College, Dhaka, BGD; 4 Medicine and Surgery, Dhaka Medical College and Hospital, Dhaka, BGD

**Keywords:** plasmapheresis, conjugated hyperbilirubinemia, acute renal failure, severe leptospirosis, therapeutic plasma exchange, weil's disease, zoonotic infection, refractory leptospirosis

## Abstract

Leptospirosis, a zoonotic infection prevalent in Pakistan, presents diverse clinical manifestations ranging from mild flu-like symptoms to severe multiorgan failure known as Weil’s disease. This case study reports on a 24-year-old woman with leptospirosis complicated by acute kidney injury and hyperbilirubinemia, unresponsive to standard therapies. Despite initial treatment with antibiotics and hemodialysis, her condition deteriorated. Following a single session of plasmapheresis, marked clinical and laboratory improvements were observed. Notably, plasma exchange effectively reduced bilirubin levels, underscoring its potential benefit in severe leptospirosis. This case highlights the role of plasmapheresis as rescue therapy in critically ill patients, demonstrating significant outcomes in cases resistant to conventional management. Further research is warranted to refine guidelines on the optimal timing and frequency of plasma exchange in such settings.

## Introduction

Leptospirosis is a disease transmitted from animals to humans (zoonotic). The causative agent is carried by various animals, both domestic and wild, such as pigs, cattle, dogs, and rodents. Leptospirosis spreads to humans through direct contact with infected animal tissues or body fluids, or indirectly through environmental exposure where the pathogen can enter through skin or mucous membrane breaches. The pathogenic bacterium *Leptospira interrogans *consists of over 250 serovars grouped into 23 serogroups [1]. Leptospirosis is prevalent in Pakistan, with an endemicity rate of 44% [2].

The range of clinical manifestations of leptospirosis varies from asymptomatic infection to severe cases characterized by septic shock and extensive multiorgan failure. Weil's disease is a severe form of leptospirosis. It is characterized by more severe clinical manifestations and can be life-threatening if not treated promptly. Leptospirosis advances through two distinct stages. Initially, there is the 'infective' or 'septicemic' phase, which typically spans four to seven days. During this period, leptospires can be identified in both the bloodstream and cerebrospinal fluid. Symptoms observed during this phase are nonspecific and can include fever, headache, muscle pain, abdominal discomfort, and inflammation of the uvea. Following this initial phase, there is a brief period lasting one to three days during which the fever diminishes. Subsequently, the 'immune' phase commences, characterized by the excretion of leptospires through the urine and the emergence of immunoglobulin M (IgM) antibodies against *Leptospira *in the bloodstream. Patients frequently experience a recurrence of initial symptoms during this phase, which may include fever and occasionally aseptic meningitis. Around 5% to 10% of patients advance to the severe manifestation of the illness, recognized as Weil's disease. This severe variant is distinguished by jaundice caused by hepatocellular dysfunction rather than liver necrosis, in addition to the involvement of multiple organs such as acute kidney injury (AKI), pulmonary bleeding, and adult respiratory distress syndrome [3].

Treating Weil’s disease requires the use of antimicrobial medications in conjunction with supportive care. This supportive care encompasses maintaining adequate hydration, offering advanced respiratory support, and quickly starting renal replacement therapy (RRT). In severe instances of Weil's disease that is resistant to standard antibiotic treatment and RRT, further therapeutic measures may be required [4].

Case studies have highlighted favorable results from employing plasmapheresis in severe instances of leptospirosis (Weil's disease). Here, we present the case of a 24-year-old woman who exhibited impaired renal function tests and elevated conjugated bilirubin levels, initially unresponsive to antimicrobial treatment but showing notable improvement following plasmapheresis.

## Case presentation

A 24-year-old married woman presented to the emergency department with a five-day history of jaundice, fever, loss of appetite, and generalized body aches. She reported no recent travel, antibiotic use, or contact with sick individuals but mentioned frequently washing clothes in the public canal. She did not experience any urinary symptoms, abdominal pain, vomiting, respiratory issues, or changes in bowel habits. Her medical, surgical, and family histories were also unremarkable.

During the examination, her vital signs included a temperature of 101°F, a pulse rate of 110 beats per minute, a blood pressure reading of 120/70 mmHg, and a respiratory rate of 19 breaths per minute. She was alert and oriented. The physical examination revealed scleral icterus and jaundiced skin. However, her abdominal, respiratory, and neurological examinations were normal.

Upon arrival, her lab results showed direct hyperbilirubinemia, abnormal renal function tests, mild hyponatremia, and hypokalemia (Table 1). She was clinically diagnosed with leptospirosis. Treatment was initiated with IV ceftriaxone 1g twice daily, oral doxycycline 100mg twice daily, and supportive care. Her virology profile, dengue serology, malaria parasite tests, coagulation profile, ECG, echocardiogram, chest X-ray, ultrasonography, urinalysis, and brucella serology were all unremarkable. Leptospira serology tested positive for IgM.

**Table 1:** All investigations during hospital stay WBC: white blood cell count; ALT: alanine transaminase; ALP: alkaline phosphatase; mcL: microliter; g/dL: gram/deciliter; mg/dL: milligram per deciliter; IU/L: international units per liter; mEq/L: milliequivalents per liter

Labs	Reference range	Day 1	Day 2	Day 3	Day 4	Day 5	Day 6	Day 7	Day 8
WBC (x10^3^/mcL)	04-11	13	N/A	N/A	09	N/A	N/A	N/A	N/A
Hemoglobin (mg/dL)	11.5-17.5	13.1	N/A	N/A	12.8	N/A	N/A	N/A	N/A
Platelet count (x10^3^/mcL)	150-450	255	N/A	N/A	278	N/A	N/A	N/A	N/A
Blood urea (mg/dL)	18-45	187	210	198	298	210	155	120	67
Creatinine (mg/dL)	0.42-1.06	2.9	4.5	4.2	8.3	4.7	3.1	2.5	1.7
Total bilirubin (mg/dL)	01-1.0	20.3	22.6	21.7	28.1	15.2	8.9	4.3	2.1
Direct bilirubin(mg/dL)	<0.3	15.5	15.9	15.1	23.7	12.5	6.2	3.1	1.6
ALT (IU/L)	10-50	130	133	N/A	N/A	N/A	N/A	38	N/A
ALP (IU/L)	35-104	210	214	N/A	N/A	N/A	N/A	55	N/A
Sodium (mEq/L)	135-151	128	121	125	132	138	134	136	137
Potassium (mEq/L)	3.5-5.1	3.1	3.3	3.2	3.5	3.7	3.4	3.8	3.7
Chloride (mEq/L)	96-112	93	91	101	98	105	102	107	105
Random blood sugar (mg/dL)	70-140	102	N/A	N/A	N/A	N/A	N/A	N/A	N/A

On the second day, we initiated one session of hemodialysis due to the increasing trend in blood urea nitrogen (BUN). However, by the third and fourth days, the patient's bilirubin levels and renal function tests (RFTs) had worsened, and her clinical condition deteriorated despite ongoing treatment. Consequently, we scheduled her for plasma exchange and performed one session of plasmapheresis on the fifth day. Following the procedure, her clinical condition and laboratory values improved significantly. By the eighth day, she had made substantial progress and was discharged with a follow-up scheduled for two weeks. After two weeks, she was in good health, with normal RFTs and bilirubin levels.

## Discussion

Leptospirosis is a prevalent zoonotic infection in Pakistan [2]. Various wild and domestic animals serve as reservoirs for *Leptospira*, with the primary reservoir for human transmission being the brown rat (*Rattus norvegicus*). The pathogen resides in the kidney tubules of these animals and is expelled through their urine. Humans contract the infection by coming into contact with contaminated urine on mucous membranes or through broken skin. The incubation period typically lasts from five to seven days [5]. Our patient frequently exposed herself to contaminated water while washing clothes in a public canal. Once inside the human body, the disease progresses through two phases: the septicemic phase and the immune phase. The septicemic phase is characterized by a sudden onset of fever, joint pain, and muscle aches, with *Leptospira *detectable in the bloodstream. Following this, there is a short period where symptoms temporarily subside before entering the immune phase. During this subsequent phase, the body's humoral response works to eliminate the organism from various tissues. However, immune complex deposition in this phase can lead to endothelial damage [6].

The primary cause of kidney damage in leptospirosis is tubulointerstitial nephritis, characterized by interstitial edema and infiltration of cells. This condition commonly presents as non-oliguric AKI, accompanied by hypokalemia due to dysfunction of the renal tubules [7]. Jaundice in leptospirosis results from pathogenic leptospires infiltrating the intercellular junctions of host hepatocytes. This infiltration disrupts these junctions, causing bile to leak from the bile canaliculi, which leads to the immediate onset of jaundice [8]. Our patient exhibited non-oliguric AKI and conjugated hyperbilirubinemia upon presentation.

Severe leptospirosis is typically treated with intravenous benzylpenicillin at 1.5 million units every six hours, ceftriaxone at 1-2g once daily, or cefotaxime at 1g every six hours, all administered for a period of seven days. Additionally, oral doxycycline at 100mg twice daily is prescribed for the same duration to complete the treatment regimen. Besides antimicrobial therapy, supportive care such as hemodialysis or RRT is essential for patients with fulminant leptospirosis who develop sepsis and acute renal failure [9]. RRT in patients with leptospirosis is effective in eliminating the inflammatory cytokines generated in response to the spirochete [10]. Our patient received IV ceftriaxone at a dose of 2g daily and doxycycline 100mg twice daily. Despite undergoing one session of hemodialysis, her clinical status and laboratory findings did not show any signs of improvement. Microcirculatory abnormalities and biliary obstruction in leptospirosis lead to impaired bilirubin excretion, resulting in elevated bilirubin levels that exceed aminotransferase levels. These elevated bilirubin levels can induce toxic effects on renal tubules, resulting in AKI. Therefore, the rapid clinical improvement observed in severe leptospirosis with plasma exchange is attributed to the removal of bilirubin [11]. Our case responded significantly to one session of plasmapheresis. There are multiple case reports that showed significant responses to plasma exchanges [11-13]. A case series involving 114 patients diagnosed with leptospirosis complicated by pulmonary hemorrhage compared outcomes between two groups: one receiving two sessions of plasma exchange plus a single dose of cyclophosphamide, and another receiving only supportive treatment. The survival rate was significantly higher among patients who underwent plasma exchange, at 77%, compared to 17% among those receiving supportive treatment alone [5].

As part of our treatment strategy for Weil's disease, plasmapheresis was used as a last-resort therapy for cases unresponsive to conventional critical care. This decision was made after thoroughly assessing the potential advantages and risks for each individual patient. The decision to initiate therapeutic plasma exchange (TPE) primarily relied on monitoring bilirubin levels, although there are no established criteria or evidence-based guidelines governing the timing, frequency, or duration of plasma exchange sessions [14]. Mild hyperbilirubinemia is typically defined as a total serum bilirubin level exceeding 5mg/dL. Although this criterion is not a definitive guideline, it is often considered as a threshold for initiating TPE. The quantity of TPE sessions provided is customized according to the patient's specific clinical progress. The optimal goal for reducing bilirubin levels following TPE remains unclear. Previous research has shown diverse outcomes, with reductions of 46.53%, 21.20%, and 37.69% observed in bilirubin levels after TPE [15].

TPE is not recommended for patients who are hemodynamically unstable, have allergies to replacement colloids/albumin, fresh frozen plasma, or heparin, or lack access to a central line or large peripheral veins. Relative contraindications include recent use of angiotensin-converting enzyme (ACE) inhibitors and hypocalcemia. Common complications associated with TPE, either during or after the procedure, are hypocalcemia, fluid and electrolyte imbalances, transfusion reactions, bleeding due to low fibrinogen levels (hypofibrinogenemia), a reduced platelet count (thrombocytopenia), and hypotension [16].

## Conclusions

Leptospirosis, a significant zoonotic infection prevalent in Pakistan, manifests through a spectrum of clinical stages and complications. This case highlights the importance of prompt diagnosis and comprehensive management in mitigating severe outcomes, such as renal failure and hepatocellular dysfunction. Initial treatment with broad-spectrum antibiotics and supportive care, including RRT when indicated, forms the cornerstone of management. In refractory cases, TPE emerges as a promising intervention, particularly for reducing bilirubin levels and improving clinical outcomes. Despite challenges in defining precise criteria for initiating and managing TPE, its role as a rescue therapy in severe cases of leptospirosis, resistant to standard care, highlights its potential benefits. Future studies aimed at refining guidelines for TPE initiation and optimization could further enhance treatment strategies for this challenging infectious disease.
